# Effective components of *Panax quinquefolius* and *Corydalis tuber* protect the myocardium by inhibiting platelet activation and improving the hypercoagulable state

**DOI:** 10.3892/etm.2015.2271

**Published:** 2015-02-06

**Authors:** MEI XUE, MEI-LIN LIU, XIN-YUAN ZHU, DA-ZHUO SHI, HUI-JUN YIN

**Affiliations:** 1 Cardiovascular Center, Xiyuan Hospital, China Academy of Chinese Medical Sciences, Haidian, Beijing 100091, P.R. China; 2 Department of Geriatric Medicine, Peking University First Hospital, Beijing 100034, P.R. China

**Keywords:** acute myocardial infarction, *Panax quinquefolius*, *Corydalis tuber*, platelet activation, hypercoagulable state

## Abstract

The aim of the present study was to investigate the effects of extract of *Panax quinquefolius* and *Corydalis tuber* (EPC) on platelet activation and the hypercoagulable state in rats with acute myocardial infarction (AMI). The MI model in Wistar rats was induced by coronary artery ligation. Sham surgery was performed as a control. The surviving rats that underwent MI surgery were divided into control (administered normal saline), metoprolol (9 mg/kg) and low-, moderate- and high-dose EPC groups (0.54, 1.08 g/kg and 2.16 g/kg, respectively). Saline, metoprolol and EPC were administered by gastrogavage for two consecutive weeks. The morphological changes of the myocardium were assessed by hematoxylin and eosin and nitroblue tetrazolium staining. Serum von Willebrand factor (vWF), D-dimer (DD), platelet membrane glycoproteins IIb-IIIa (GPIIb-IIIa) and CD62P levels were assessed using enzyme-linked immunosorbent assay. EPC attenuated the pathological changes of the myocardium. High-dose EPC decreased the serum concentration of vWF when compared with control group. Moderate and high doses of EPC decreased the DD and GPIIb-IIIa levels, and the CD62P level was gradually decreased with EPC dose escalation. The results therefore demonstrated that EPC protects the myocardium by inhibiting platelet activation and improving the hypercoagulable state in a rat model of AMI.

## Introduction

Extract of *Panax quinquefolius* and *Corydalis tuber* (EPC), which is primarily composed of *P. quinquefolius* saponins and tetrahydropalmatine, has previously shown efficacy in the treatment of ischemic cardiovascular diseases in the clinic (1). In Traditional Chinese Medicine, *P. quinquefolius* is known to invigorate Qi and nourish Yin, clear fire and generate body fluid. *C. tuber*, which has an acrid taste, activates blood circulation and regulates Qi to alleviate pain (2,3). These two ingredients accentuate each other. Our recent study revealed that, following myocardial infarction (MI), EPC exerted significant protective effects against oxidative stress injury in the myocardium by increasing superoxide dismutase activity and decreasing levels of 8-iso-prostaglandin F2α (1). Furthermore, moderate-to-high doses of EPC significantly decreased the mRNA and protein expression of 78-kDa glucose-regulated protein and C/EBP-homologous protein when compared with the control group, indicating that EPC could alleviate injury to the myocardium following MI by suppressing excessive endoplasmic reticulum stress (1). In another study, EPC treatment significantly inhibited ERS and oxidatedive stress, balanced the Bcl-2/bax ratio, suppressed the activation of caspase-3 and exerted anti-apoptotic effects in pigs with larger anterior wall AMI (4).

Abnormalities in the coagulation and fibrinolytic system and platelet activation are the principal pathophysiological features of coronary heart disease, particularly for MI (5). The aim of the present study was to investigate the hypothesis that EPC acts to protect against MI by inhibiting platelet activation and improving the hypercoagulable state, in order to elucidate part of the pharmacological mechanism of EPC.

## Materials and methods

### Animals

One hundred male Wistar rats, weighing 180±20 g, were purchased from the Institute of Laboratory Animal Sciences, Chinese Academy of Medical Sciences (certificate no. SCXK Beijing 2005–0013; Beijing, China). All rats were housed in humidity-controlled rooms (55±5%) at 22±2°C with a 12-h light/dark cycle and were fed standard rat chow. All animals were cared for in accordance with the policies and guidelines released by the Animal Care and Ethics Committee of the China Academy of Chinese Medical Sciences (Beijing, China).

### Preparation of EPC

EPC was provided by the Institute of Chinese Materia Medica, China Academy of Chinese Medical Sciences. The main active components of the extract are shown in Table I, as measured by the high-performance liquid chromatography (HPLC) method (1,6). The main active components of the extract were separated using a Kromasil 100-5C_18_ column (4 μml 4.5×150 mm; EKA Nobel, Bohus, Sweden) at a flow rate of 1.0 μl/l. Gradient elution was performed, at a ratio of 81:19 (A:B, v/v, A:0.01 mol/l sodium dihydrogen phosphate and disodium gydrogen phosphate, B: acetonitrile).

### Animal model establishment and grouping

Ten rats were randomly selected as a sham group, and the remaining rats were randomly divided into five groups: Control, metoprolol, low-dose EPC, moderate-dose EPC and high-dose EPC (n=18/group). Anesthesia was induced with an intraperitoneal injection of urethane solution (20%) at a dose of 0.6 ml/kg. The left anterior descending coronary artery (LAD) was ligated to establish the MI model according to the methods of Olivetti, as described previously (7–9). The rats in the sham group did not undergo ligation.

### Treatment methods

Following the MI surgery, group-specific treatments were given to the surviving rats. The metoprolol group was administered metoprolol (9 mg/kg; batch no. 1012055; AstraZeneca Pharmaceutical Co., Ltd., London, UK) and the low-, moderate- and high-dose EPC groups were administered EPC at doses of 0.54, 1.08 and 2.16 g/kg, respectively, by gastrogavage once every 24 h for two weeks. An equal volume of normal saline was given to the sham and control groups. The rats were sacrificed at the end of the 2 week period using intraperitoneal injection of 20% urethane

### Pathomorphological analysis

Two left ventricular myocardia from each group were selected randomly following blood collection, fixed in 10% neutral formalin buffer and paraffin-embedded subsequent to dehydration to carry out the hematoxylin and eosin (HE) staining. The other ischemic hearts were rinsed in normal saline to remove the blood, cut into four pieces parallel to the coronary sulcus, and then incubated in 10% nitroblue tetrazolium (NBT; 50 mg/100 ml; Sigma, St. Louis, MO, USA) at 37°C for 10 min. The infarct size was quantified using Image Pro Plus software (version 4.0; Media Cybernetics, Inc., Rockville, MD, USA), and was expressed as the proportion of infarct in the left ventricular.

### Enzyme-linked immunosorbent assay (ELISA) for the detection of serum levels of von Willebrand factor (vWF), D-dimer (DD), platelet membrane glycoproteins IIb-IIIa (GPIIb-IIIa) and CD62P

The serum levels of vWF, DD, GPIIb-IIIa and CD62P were detected using ELISA with kits provided by the Sino-American Biotechnology Co., Ltd. (Wuhan, China), according to the manufacturer’s instructions. A Multiskan™ MK3 microplate reader (Thermo Fisher Scientific, Inc., Waltham, MA, USA) was used for the detection.

### Statistical analysis

Data from at least nine independent experiments are presented as the mean ± standard deviation. One-way analysis of variance was performed for the comparison of the means. All statistical analyses were carried out with SPSS software (version 11.0; SPSS, Inc., Chicago, IL USA), and P<0.05 were considered to indicate a statistically significant difference.

## Results

### General observations

Twenty-four hours after LAD ligation, the surviving rats with ST segment elevation (monitored by lead II of the electrocardiogram) included nine rats in the control group, 12 in the metoprolol group, nine in the low-dose EPC group, 11 in the moderate-dose EPC group and 10 in the high-dose EPC group, in addition to the 10 rats in the sham group. The rats in the different groups exhibited normal physical appearance and behavior during the two-week gavage period.

### Morphological changes in the myocardium among the groups

**Figure 1:**
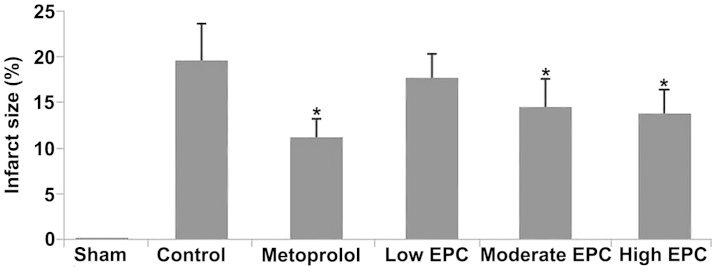
Metoprolol and EPC administration decrease myocardial infarct size. Results are presented as the mean ± standard deviation. ^*^P<0.05, vs. the control group. EPC, extract of *Panax quinquefolius* and *Corydalis tuber*.

NBT stained the normal myocardium dark blue, while the infarcted myocardium exhibited no staining. The myocardial infarct size was larger in the control group when compared with that in the sham group (Fig. 1). Furthermore, the myocardial infarct size was decreased in the metoprolol and low-, moderate- and high-dose EPC groups when compared with that in the control group (Figs. 1).

**Figure 2:**
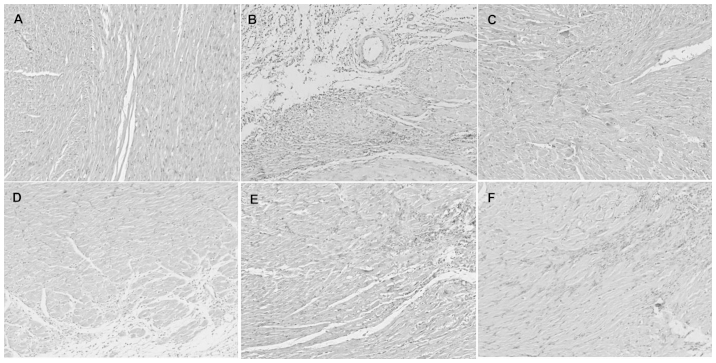
Comparison of myocardial infarct size in different groups (hematoxylin and eosin staining; magnification, ×100): (A) Sham; (B) control; (C) metoprolol; (D) low-dose EPC; (E) moderate-dose EPC; (F) high-dose EPC. EPC, extract of *Panax quinquefolius* and *Corydalis tuber*.

HE staining showed that the myocardial cells of the sham group were arranged in an orderly manner, with normal morphology and texture (Fig. 2A). The myocardial cells in the control group exhibited a swollen appearance and formed a wave shape with vacuolar degeneration and fibrosis (Fig. 2B). These changes in cardiac structure were significantly attenuated in the metoprolol group (Fig. 2C) and with all doses of EPC (Fig. 2D–F for the low-, medium- and high-dose groups, respectively).

### Expression of vWF and DD in the serum

Compared with levels in the sham group, the serum vWF and DD levels in the control group were significantly increased (P<0.01, Table II). Metoprolol and high-dose EPC decreased the serum concentration of vWF when compared with the control group (P<0.01). Moderate- and high-dose EPC decreased the DD level in serum when compared with the control group (P<0.05 and P<0.01, respectively).

### Expression of GPIIb-IIIa and CD62P in the serum

Compared with levels in the sham group, the serum GPIIb-IIIa and CD62P levels in the control group were significantly increased (P<0.05, Table III). Moderate and high doses of EPC decreased the GPIIb-IIIa level when compared with the control group (P<0.01). EPC reduced the CD62P serum level gradually at all doses, with dose escalation (P<0.05 and P<0.01).

## Discussion

Platelet activation is a key pathophysiological feature of coronary heart disease, particularly for MI (10). The formation of a platelet plug at sites of atherosclerotic lesion rupture is the most common mechanism leading to MI (11). In the present study the LAD was ligated to establish an MI model. The results showed that the serum GPIIb-IIIa and CD62P levels in the MI rats were significantly increased compared with those in the sham group. Since GPIIb-IIIa plays an essential role in platelet aggregation and CD62P is the marker of late-phase platelet activation, and can only be expressed on the degranulated platelet surface (12,13), these proteins are specific indices presently known to be able to most directly reflect the degree of platelet activation (14,15). The present results therefore indicated that the platelets were activated in the rats following MI.

Abnormalities in the coagulation and fibrinolytic system have been associated with an increased risk of coronary heart disease in observational studies and meta-analyses (5,10). In the process of platelet activation, the regulation of binding between vWF and the platelet receptor GPIbα is one of the key steps for platelet adhesion (11,16,17). The present results showed that the serum vWF level was increased in the MI model when compared with that in the sham group, which indicated an enhancement of platelet adhesion and thrombogenesis. Fibrin DD is the primary degradation product of cross-linked fibrin and is a marker of activated coagulation and fibrinolysis (18). The rise in DD at the early stage of acute MI (AMI) may be useful to indicate the critical condition of patients with AMI (19). The present results showed that the serum vWF and DD levels in the control group were significantly increased compared with those in the sham group, indicating that, subsequent to MI, a hypercoagulable state was induced in the rat model, followed by platelet activation, thrombosis and subsequent fibrinolysis. This may explain the severe pathological damages in the control group, as shown by NBT and HE staining.

*P. quinquefolius* has the effects of invigorating Qi and nourishing Yin, clearing fire and generating body fluid. *C. tuber*, which has an acrid taste, activates blood circulation and regulates Qi to alleviate pain, as stated in the Chinese Pharmacopoeia (3). EPC, an extract of *P. quinquefolius* and *C. tuber*, has long been used for the treatment of ischemic cardiovascular diseases in the clinic. *P. quinquefolius* saponins and tetrahydropalmatine are the active components of EPC, as determined by HPLC. Previous animal experiments and clinical trials have found that *P. quinquefolius* saponins possess varied pharmacological properties, and exert anti-anoxia, anti-ischemic and antioxidation effects (4,20). Furthermore, tetrahydropalmatine has been demonstrated to have analgesic and sedative effects (21). The present results showed that high-dose EPC decreased the serum concentration of vWF when compared with the control group. Moderate and high doses of EPC decreased the DD and GPIIb-IIIa levels, and the CD62P levels were gradually decreased with EPC dose escalation. It can therefore be concluded from the findings with this rat model of AMI that EPC has a therapeutic role in inhibiting platelet activation and improving the hypercoagulable state by suppressing the expression of GPIIb-IIIa, CD62P, vWF and DD.

Metoprolol, which is a key drug in the therapy of post-infarct hearts, has an important effect in decreasing mortality in patients following AMI (22). Metoprolol was thus used as the control drug in the present study. Infarct size predicts post-infarction mortality (22). In this study, NBT and HE staining showed that metoprolol and EPC reduced myocardial infarct size, and metoprolol decreased the serum concentration of vWF when compared with control group; however, metoprolol showed no effects on platelet activation and oxidative injury. We therefore speculated that the cardioprotective mechanisms of metoprolol were achieved predominantly by blocking cardiac β1-receptors and thereby slowing the heart rate and reducing myocardial contractility and oxygen consumption, as previously reported (23). Therefore, EPC has a therapeutic effect in a rat model of AMI by attenuating the pathological changes of the myocardium, inhibiting platelet activation and improving the hypercoagulable state.

## Figures and Tables

**Table I:** Quality evaluation of extract of *Panax quinquefolius* and *Corydalis tuber*.

Major constituent	Content (%)
Ginsenoside Rg1	0.11
Ginsenoside Re	1.88
Ginsenoside Rb1	5.30
Tetrahydropalmatine	0.07

**Table II:** Comparison of the levels of serum vWF and DD among the groups.

Group	n	vWF (ng/ml)	DD (ng/ml)
Sham	10	4549.75±844.76^b^	24.67±8.64^b^
Control	9	6163.22±1045.94	55.62±15.42
Metoprolol	12	4621.94±1002.79^b^	45.36±15.15
Low-dose EPC	9	5624.18±1034.12	43.21±13.06
Moderate-dose EPC	11	5672.15±965.41	25.85±4.60^b^
High-dose EPC	10	4093.56±977.52^b^	32.30±10.28^a^

Results are presented as the mean ± standard deviation.

a P<0.05 and

b P<0.01, vs. the control group.

EPC, extract of *Panax quinquefolius* and *Corydalis tuber*; vWF, von Willebrand factor; DD, D-dimer.

**Table III:** Comparison of the levels of serum GPIIb-IIIa and CD62P among the groups.

Group	n	GPIIb-IIIa (ng/ml)	CD62P (ng/ml)
Sham	10	5.31±1.06^a^	31.28±8.92^a^
Control	9	7.71±1.16	51.57±9.52
Metoprolol	12	5.56±1.05	34.24±11.35
Low-dose EPC	9	6.97±1.13	30.55±12.76^a^
Moderate-dose EPC	11	3.91±1.11^b^	25.52±10.60^b^
High-dose EPC	10	4.59±1.08^b^	23.26±14.16^b^

Results are presented as the mean ± standard deviation.

a P<0.05 and

b P<0.01, vs. the control group.

EPC, extract of *Panax quinquefolius* and *Corydalis tuber*; GPIIb-IIIa, glycoproteins IIb-IIIa.
